# TRAIT: A Comprehensive Database for T-cell Receptor–antigen Interactions

**DOI:** 10.1093/gpbjnl/qzaf033

**Published:** 2025-04-21

**Authors:** Mengmeng Wei, Jingcheng Wu, Shengzuo Bai, Yuxuan Zhou, Yichang Chen, Xue Zhang, Wenyi Zhao, Ying Chi, Gang Pan, Feng Zhu, Shuqing Chen, Zhan Zhou

**Affiliations:** State Key Laboratory of Advanced Drug Delivery and Release Systems & Zhejiang Provincial Key Laboratory of Anti-Cancer Drug Research, College of Pharmaceutical Sciences, Zhejiang University, Hangzhou 310058, China; Collaborative Innovation Center of Artificial Intelligence by MOE and Zhejiang Provincial Government, College of Computer Science and Technology, Zhejiang University, Hangzhou 310027, China; State Key Laboratory of Advanced Drug Delivery and Release Systems & Zhejiang Provincial Key Laboratory of Anti-Cancer Drug Research, College of Pharmaceutical Sciences, Zhejiang University, Hangzhou 310058, China; State Key Laboratory of Advanced Drug Delivery and Release Systems & Zhejiang Provincial Key Laboratory of Anti-Cancer Drug Research, College of Pharmaceutical Sciences, Zhejiang University, Hangzhou 310058, China; State Key Laboratory of Advanced Drug Delivery and Release Systems & Zhejiang Provincial Key Laboratory of Anti-Cancer Drug Research, College of Pharmaceutical Sciences, Zhejiang University, Hangzhou 310058, China; State Key Laboratory of Advanced Drug Delivery and Release Systems & Zhejiang Provincial Key Laboratory of Anti-Cancer Drug Research, College of Pharmaceutical Sciences, Zhejiang University, Hangzhou 310058, China; State Key Laboratory of Advanced Drug Delivery and Release Systems & Zhejiang Provincial Key Laboratory of Anti-Cancer Drug Research, College of Pharmaceutical Sciences, Zhejiang University, Hangzhou 310058, China; State Key Laboratory of Advanced Drug Delivery and Release Systems & Zhejiang Provincial Key Laboratory of Anti-Cancer Drug Research, College of Pharmaceutical Sciences, Zhejiang University, Hangzhou 310058, China; Innovation Institute for Artificial Intelligence in Medicine & MOE Engineering Research Center of Innovative Anticancer Drugs, Zhejiang University, Hangzhou 310018, China; ZJU-UoE Institute, Zhejiang University, Haining 314400, China; Alibaba-Zhejiang University Joint Research Center of Future Digital Healthcare, Hangzhou 310058, China; Collaborative Innovation Center of Artificial Intelligence by MOE and Zhejiang Provincial Government, College of Computer Science and Technology, Zhejiang University, Hangzhou 310027, China; State Key Laboratory of Advanced Drug Delivery and Release Systems & Zhejiang Provincial Key Laboratory of Anti-Cancer Drug Research, College of Pharmaceutical Sciences, Zhejiang University, Hangzhou 310058, China; Innovation Institute for Artificial Intelligence in Medicine & MOE Engineering Research Center of Innovative Anticancer Drugs, Zhejiang University, Hangzhou 310018, China; Alibaba-Zhejiang University Joint Research Center of Future Digital Healthcare, Hangzhou 310058, China; State Key Laboratory of Advanced Drug Delivery and Release Systems & Zhejiang Provincial Key Laboratory of Anti-Cancer Drug Research, College of Pharmaceutical Sciences, Zhejiang University, Hangzhou 310058, China; State Key Laboratory of Advanced Drug Delivery and Release Systems & Zhejiang Provincial Key Laboratory of Anti-Cancer Drug Research, College of Pharmaceutical Sciences, Zhejiang University, Hangzhou 310058, China; Innovation Institute for Artificial Intelligence in Medicine & MOE Engineering Research Center of Innovative Anticancer Drugs, Zhejiang University, Hangzhou 310018, China; Alibaba-Zhejiang University Joint Research Center of Future Digital Healthcare, Hangzhou 310058, China; The Fourth Affiliated Hospital, Zhejiang University School of Medicine, Yiwu 322000, China

**Keywords:** T-cell receptor–antigen interaction, Antigen recognition, Mutation, Single-cell omics, T-cell-based immunotherapy

## Abstract

Comprehensive and integrated resources on interactions between T-cell receptors (TCRs) and antigens are still lacking for adoptive T-cell-based immunotherapies, highlighting a significant gap that must be addressed to fully understand the mechanisms of antigen recognition by T cells. In this study, we present the T-cell receptor–antigen interaction database (TRAIT), a comprehensive database that profiles the interactions between TCRs and antigens. TRAIT stands out due to its comprehensive description of TCR–antigen interactions by integrating sequences, structures, and affinities. It provides millions of experimentally validated TCR–antigen pairs, resulting in an exhaustive landscape of antigen-specific TCRs. Notably, TRAIT emphasizes single-cell omics as a major reliable data source for TCR–antigen interactions and includes millions of reliable non-interactive TCRs. Additionally, it thoroughly demonstrates the interactions between mutations of TCRs and antigens, thereby benefiting affinity optimization of engineered TCRs as well as vaccine design. TCRs on clinical trials are innovatively provided. With the significant efforts made toward elucidating the complex interactions between TCRs and antigens, TRAIT is expected to ultimately contribute superior algorithms and substantial advancements in the field of T-cell-based immunotherapies. TRAIT is freely accessible at https://pgx.zju.edu.cn/traitdb.

## Graphical abstract

**Figure:**
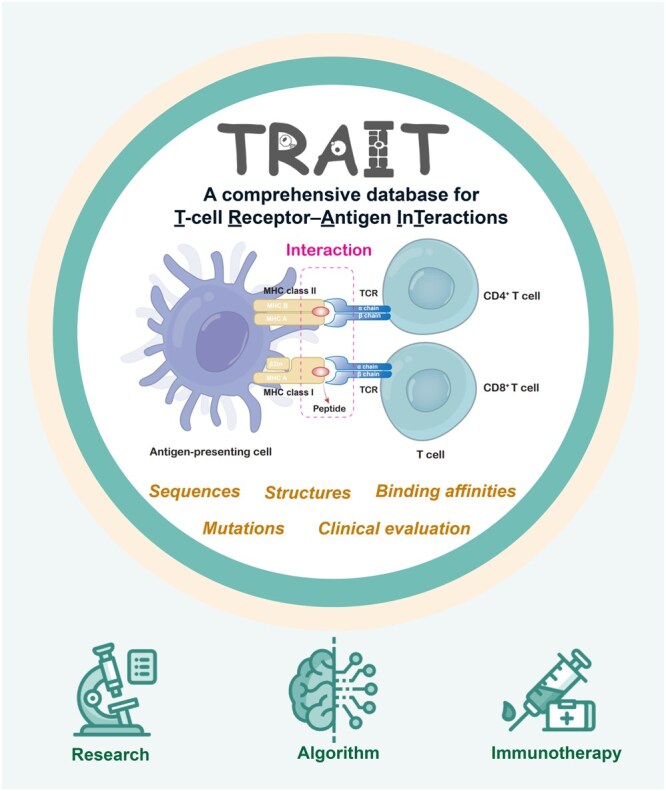

## Introduction

Remarkable T-cell receptors (TCRs) play a vital role in initiating intracellular signals required for the effector response to foreign antigens in the adaptive immune system [1,2]. TCR provides the initial signal for T-cell activation by mediating the specific recognition of pathogen-derived epitopes through its interaction with the peptide–major histocompatibility complex (pMHC) multimer [3–5]. The underlying basis for the exceptional sensitivity and discriminatory recognition lies in the hypervariable complementarity-determining region 3 (CDR3) loops of TCRs, which interact mainly with the peptide, whereas the CDR1 and CDR2 regions contact the MHC α-helices [6,7]. The vast diversity of CDR3 regions and the cross-reactivity of TCRs ensure that the available TCR repertoire can recognize a wide range of pathogen-derived epitopes encountered over a lifetime. Nevertheless, the rules of how TCRs recognize cognate antigens (pMHCs) remain poorly understood [8,9]. Therefore, a precise and thorough characterization of the interactions between TCRs and antigens is important for understanding the molecular mechanisms of pathogen elimination and tumor surveillance mediated by adaptive immune responses.

Owing to their great importance, data including sequences, structures, and specificities of TCRs, TCR repertoires from different donors, and affinities of TCR–antigen pairs and their mutations have attracted extensive interest [10–12]. Furthermore, these data are essential for the fields of TCR engineering and the development of immunotherapies [13–15]. To date, several TCR-related databases have been developed and are currently active [16–26], and the majority of these databases focus on providing structural data of TCRs or TCR–pMHC complexes, such as the structural T-cell receptor database (STCRDab) [16], the T cell receptor structural repertoire database (TCR3d) [17], and the database of altered TCR ligand affinities and structures (ATLAS) [18]. Others are specialized in describing sequences of TCR repertoires, including the unconventional T cell receptor sequence database (UcTCRdb) [19], the pan immune repertoire database (PIRD) [20], and the human TCR sequence database (TCRdb) [21]. Additionally, several reputable databases have demonstrated a wealth of information on TCRs with known antigen specificity, such as the manually curated catalogue of pathology-associated T cell receptor sequences (McPAS-TCR) [22], the curated database of T-cell receptor sequences with known antigen specificity (VDJdb) [23,24], the immunoinformatic database of experimentally-supported functional neoantigen-specific TCR sequences (NeoTCR) [25], and the immune epitope database (IEDB) [26]. Although databases containing valuable TCR data are available, most existing ones concentrate on a specific aspect. Binding affinity and clinical evaluation of TCRs are not typically included in the majority of active databases (**Table 1**). There is noticeable gap when it comes to an integrated database that compiles a landscape of TCR–antigen interactions, highlighting the urgent need for a comprehensive database profiling TCR–antigen interactions.

**Table 1:** An overview of databases related to TCRs

Database	Sequence	Structure	Specificity	Binding affinity	Non-interactive pair	Mutation	Clinical evaluation	Active	Ref.
STCRDab		√						√	[16]
TCR3d		√						√	[17]
ATLAS		√		√		√		No longer updated after 2017	[18]
UcTCRdb	√							√	[19]
PIRD	√							√	[20]
TCRdb	√							√	[21]
McPAS-TCR	√		√					√	[22]
VDJdb	√	√	√					√	[23]
NeoTCR	√		√					√	[25]
IEDB	√		√					√	[26]
TRAIT	√	√	√	√	√	√	√	√	This study

*Note*: TCR, T-cell receptor; STCRDab, structural T-cell receptor database; TCR3d, T cell receptor structural repertoire database; ATLAS, database of altered TCR ligand affinities and structures; UcTCRdb, unconventional T cell receptor sequence database; PIRD, pan immune repertoire database; TCRdb, human TCR sequence database; McPAS-TCR, manually curated catalogue of pathology-associated T cell receptor sequences; VDJdb, curated database of T-cell receptor sequences with known antigen specificity; NeoTCR, immunoinformatic database of experimentally-supported functional neoantigen-specific TCR sequences; IEDB, immune epitope database; TRAIT, T-cell receptor–antigen interaction database.

In this study, we developed a novel knowledge base focused on the T-cell receptor–antigen interactions (TRAIT). First, an extensive literature review was conducted by performing keyword searches in PubMed, public datasets, and omics platform, resulting in a total of 3,393,826 antigen-specificity validated TCR sequences for 1184 unique epitopes and 112 MHC alleles. Uniquely, 3,342,225 non-interactive TCR sequences verified by single-cell omics were collected and recorded. Second, structures of TCR–antigen complexes were systematically collected from the Protein Data Bank (PDB) [27], and a total of 223 structures were identified. Subsequently, valuable three-dimensional affinity data quantifying the binding ability of TCRs and antigens were manually retrieved, leading to 945 binding affinity records. Additionally, a unique dataset consisting of 641 TCR mutants and 628 antigen mutants was compiled, with each mutant linked to its respective binding affinity. Finally, TCRs with clinical evaluations were consulted exhaustively, and 34 clinical trials inferring 65 antigen-specific TCRs were identified.

In summary, TRAIT is developed to explicitly characterize the interactions between TCRs and antigens by integrating sequences, structures, and binding affinities. TRAIT also offers single-cell-based antigen-specific TCR repertoires and systematically demonstrates interactions between mutations of TCRs and antigens. Additionally, clinical evaluations of antigen-specific TCRs are uniquely provided. Given the recent prominence of T-cell-based immunotherapies in disease treatment, the comprehensive data on TCR–antigen interactions provided by TRAIT are anticipated to better facilitate the discovery and optimization of TCR candidates for immunotherapies.

## Data collection and processing

### Collection and processing of interaction data between TCRs and antigens

Based on literature evidence of TCR–antigen pairs including sequences, structures, and binding affinities, data were first collected by searching PubMed [28] for keywords with the combined terms “T cell receptor + repertoire”, “T cell receptor + affinity”, “T cell receptor + specificity”, “T cell receptor + major histocompatibility complexes”, and so on. Sequences of antigen-specific TCRs were also collected from public databases such as VDJdb [24], McPAS-TCR [22], and IEDB [26], as well as omics datasets available on the 10X website. The available raw data were downloaded and processed through IMGT/V-QUEST to produce annotated information for TCRs that lacked V and/or J specifications or had incomplete or overly extended CDR3 sequences. Structures of TCR–antigen complexes were further identified from PDB [27] with TCR–antigen reference sequences as queries. Only structures of TCR–MHC or TCR–pMHC complexes were collected and linked to their corresponding sequence entries in TRAIT. The binding affinities of TCR–antigen pairs experimentally determined using purified proteins by surface plasmon resonance (SPR) or isothermal titration calorimetry (ITC) were collected and recorded. Finally, the manually collected data of TCR–antigen pairs were further checked to remove duplicates.

### Single-cell omics-based antigen-specific TCR identification

Datasets on the 10X website that describe TCRs extracted from pMHC multimer-labeled T cells and identified by single-cell sequencing were collected and recorded as “interactive pairs” in TRAIT. Simultaneously, TCRs extracted from non-binding T-cell populations after staining with a pMHC multimer and identified by single-cell sequencing were collected and recorded as “non-interactive pairs”.

### Mutation classification and binding affinity annotation

A systemic literature review was conducted using combinations of keywords such as “T cell receptors + mutations”, “T cell receptors + variants”, “T cell receptors + directed evolution”, “T cell receptors + affinity maturation”, “major histocompatibility complex + mutations”, “major histocompatibility complex + variants”, and so on. Mutations of TCRs or antigens with binding affinities measured experimentally were collected and recorded in TRAIT. All of the entries were divided into two categories including “mutations of TCRs” and “mutations of antigens” according to the sites of mutated amino acids. CDR sequences of TCRs were annotated through IMGT/V-QUEST by inputting their full-length sequences.

### Collection of TCRs on clinical trials

A comprehensive literature review was conducted across multiple sources, including official patent organizations, various pharmaceutical company pipelines, and literature databases, using keywords “TCR-T”, “TCR-transduced T cell”, “TCR-engineered T cell”, and so on. Detailed activity data of TCR gene-engineered T cells (TCR-T) including objective response rates (ORRs), clinical response, and toxicities related to TCR-T in different stages of clinical trials were collected and displayed in TRAIT.

## Database content and construction

As a result, nearly 3.4 million records of TCR–antigen pairs associated with cancer, autoimmune diseases, and infectious diseases were collected, and the statistical data are presented in **Table 2**. Large-scale TCR–antigen pairs including sequences (3,393,826 records), structures (223 records), and binding affinities (945 records) were manually retrieved. Multiple antigen species, including *Homo Sapiens* (*n* = 3176), Influenza A virus (*n* = 9863), Epstein-Barr virus (EBV, *n* = 6227), cytomegalovirus (CMV, *n* = 22,296), and human immunodeficiency virus (HIV, *n* = 2663), were involved, and a total of 112 MHC alleles were extracted. Ultimately, a total of 20,251 interactive TCRs and 3,342,225 non-interactive TCRs referring to 50 antigens from four different donors were collected and recorded in TRAIT, and a total of 1269 mutation records (including 641 TCR mutations referring to 41 wild-type antigens and 628 antigen mutations referring to 65 wild-type TCRs) were presented. Additionally, TRAIT innovatively compiles information on 34 TCR-T cell therapies that are in clinical trial stage and have published their clinical outcomes. A total of 65 TCR–antigen pairs (59 wild-type TCRs and 6 modified TCRs) and 25 cancer types were inferred.

**Table 2:** Statistics categorized by epitope species in TRAIT

Epitope species	No. of unique epitopes	No. of interactive TCRs	No. of non-interactive TCRs	No. of structures	No. of affinity records	No. of TCR mutations	No. of antigen mutations
*Homo sapiens*	251	3176	1,343,083	81	334	232	107
Influenza A	22	9863	65,924	16	27	23	4
EBV	30	6227	803,057	15	117	65	53
CMV	32	22,296	592,849	6	12	2	10
HIV	54	2663	268,685	23	59	28	31
HPV	1	14	134,337	0	0	0	0
HCV	15	1111	0	5	6	0	6
HTLV-1	8	176	67,144	9	137	114	24
Others	771	6075	67,146	68	253	177	393
Total	1184	51,601	3,342,225	223	945	641	628

*Note*: Data were collected until March 2025. EBV, Epstein-Barr virus; CMV, cytomegalovirus; HIV, human immunodeficiency virus; HPV, human papilloma virus; HCV, hepatitis C virus; HTLV-1, human T-lymphotropic virus type 1.

**Figure 1:**
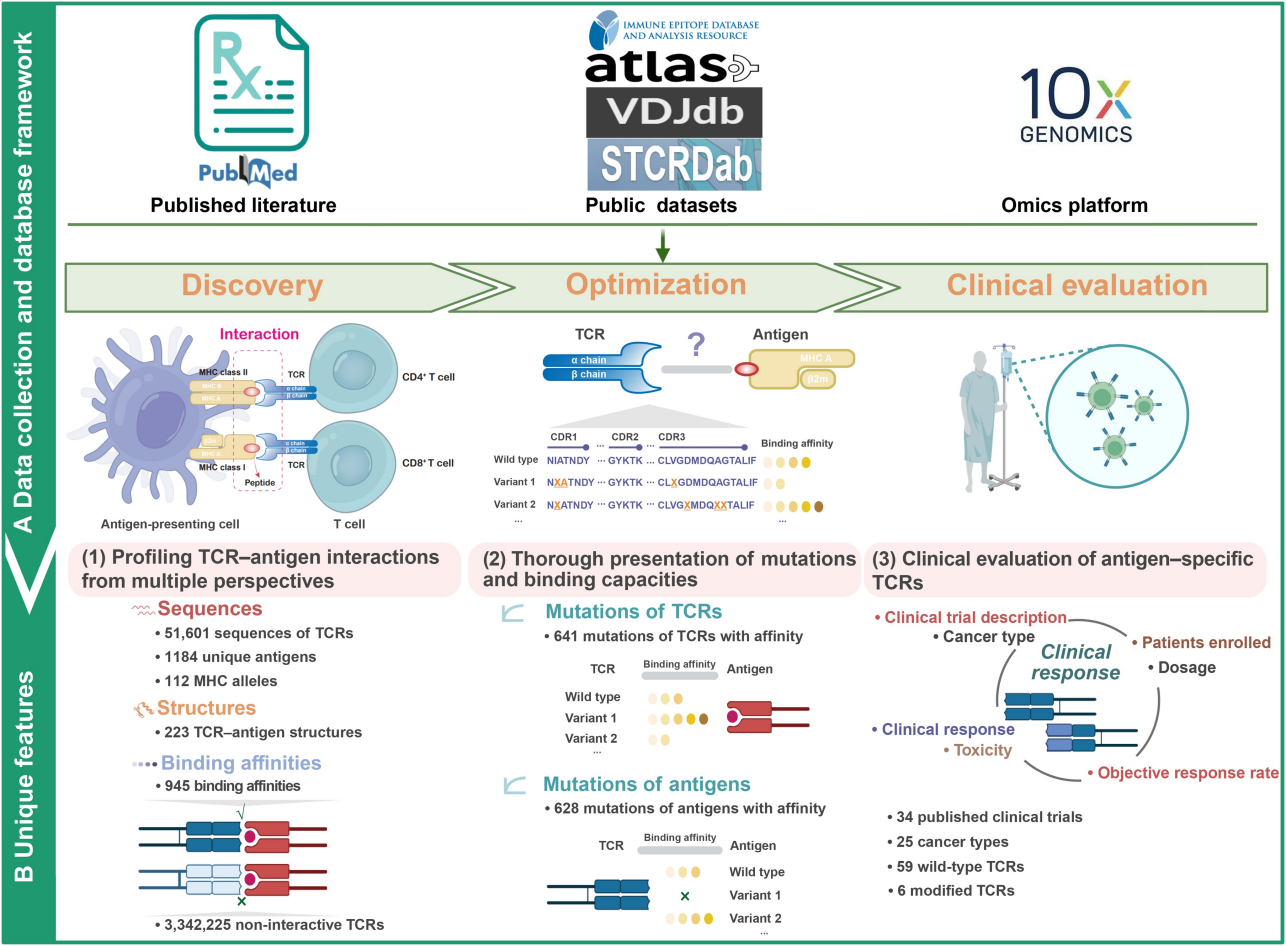
Overview of the TRAIT database **A**. Data collection and database framework. Information of antigen-specific TCRs, including sequences, structures, binding affinities, mutations with binding capacities, and clinical response, was thoroughly collected from published literature, public databases, and 10X omics platform. And then data were reorganized by the whole process of “discovery–optimization–clinical evaluation” of antigen-specific TCRs. **B**. Unique features of TRAIT. TRAIT is unique in (1) unambiguously profiling the interactions between TCRs and antigens by integrating sequences, structures, and affinities, thus offering an exhaustively landscape of TCR–antigen interactions, (2) systematically describing mutation–affinity relationship of TCRs and antigens, benefiting affinity optimization of TCRs, and (3) thoroughly demonstrating clinical response of TCRs on clinical trials. The figure is created with BioRender.com. TCR, T-cell receptor; MHC, major histocompatibility complex; CDR1/2/3, complementarity-determining region 1/2/3; β2m, beta-2 microglobulin; TRAIT, T-cell receptor–antigen interaction database.

TRAIT was built using a combination of hypertext preprocessor (PHP; https://www.php.net), Bootstrap (https://getbootstrap.com), and WordPress (https://wordpress.org) — a PHP-based blogging platform that enables users to create websites on servers supporting PHP and MySQL databases. The jQuery (https://jquery.com) and DataTables are employed to showcase the query result tables. All data in this database are stored and managed in MySQL. The overall workflow of TRAIT is depicted in **Figure 1**.

## User interface

**Figure 2:**
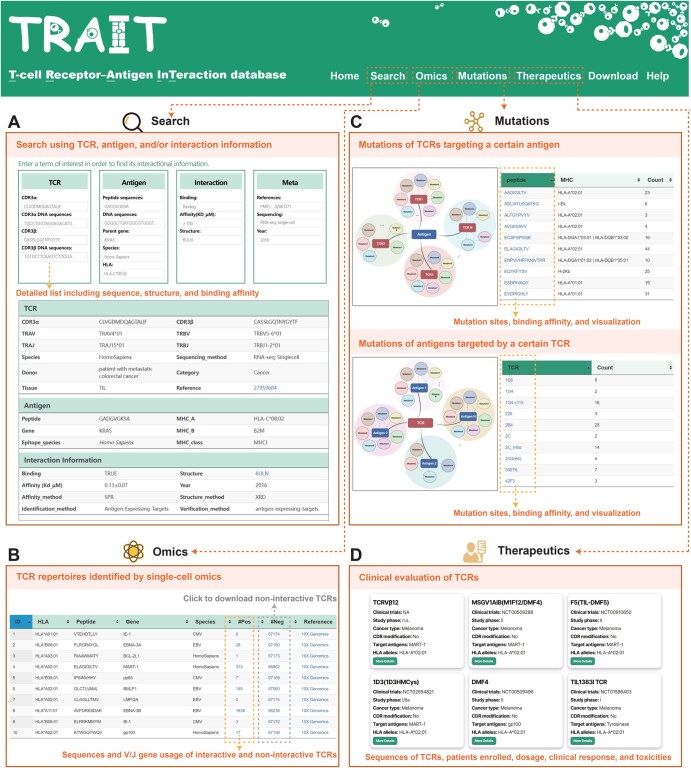
Schematic illustrations of the four primary functional pages in TRAIT **A**. The “Search” page of TRAIT. Users can browse the detailed information including sequences, structures, and binding affinities of TCR–antigen pairs. **B**. The “Omics” page of TRAIT. Validated antigen-specific TCRs including interactive and non-interactive TCRs by single-cell sequencing are displayed on the page. **C**. The “Mutations” page of TRAIT. Mutations of TCRs and antigens were classified and linked to their binding affinities on the page. Users can visualize changes in binding affinities between wild type and mutants. **D**. The “Therapeutics” page of TRAIT. The page offers detailed clinical information of TCRs on clinical trials.

TRAIT has a user-friendly web interface containing seven pages: “Home”, “Search”, “Omics”, “Mutations”, “Therapeutics”, “Download”, and “Help” (**Figure 2**). The “Home” page provides an introduction to the entire database, an overview of collected data, and a visualization of total records. The “Search” page in TRAIT provides a comprehensive repository of TCR–antigen pairs. Users can query the database using a CDR3 sequence (nucleic acid or amino acid sequence), antigen information (epitope, parent gene, species, or MHC allele), interaction information (binding affinity or structure), or metadata (reference PMID, TCR sequencing method, and publication year). A built-in search box allows users to refine results to a specific subject by entering additional search terms. The retrieved results, which include brief descriptions of the TCR–antigen pairs, are presented in a new table. Clicking on “ID” in the first column opens a results page with more detailed interaction features, including inferred sequences, structures, and binding affinities for the matching term. TCR annotations [such as CDR3α, CDR3β, V(D)J recombination, species, and sequencing methods], antigen annotations (including peptide sequences, MHC allele, gene of epitope, and species of epitope), interaction information (such as binding affinity), and experimental details (such as identification and verification methods, samples, and pathology) are also presented. More importantly, the structures of the TCR–antigen complexes were linked to the interaction information described above.

The “Omics” page offers a query of TCR–antigen pairs, including interactive and non-interactive pairs verified by single-cell sequencing. Clicking on “#Pos” in the sixth column opens a separate page for the specific antigen, showcasing detailed information about interactive TCRs and visualizations of all TCRs involved with the antigen. Users can download non-interactive TCRs for certain antigens by clicking the button “#Neg”. The “Mutations” page provides existing experimentally verified mutations of TCR–antigen pairs and their corresponding binding capacities. Clicking the TCR or antigen of interest opens a separate page for the list of all mutations and visualization of the affinity and sequence alignment. The “Therapeutics” page provides a detailed overview of the pharmaceutical information for each studied TCR in clinical status. Users may click on the “More Details” button to access more information, including sequences of TCR–antigen pairs, targeted disease indications, clinical trial status, and various clinical response data (*e.g.*, number of enrolled patients, administration dosage, ORRs, and toxicities related to the studied TCR-T cells). Users can download data and obtain tutorials from the “Download” and “Help” pages, respectively.

## Database application

### Systematic description of TCR–antigen interactions from multiple perspectives

The engagement of TCRs with antigens constitutes one of the most complex interactions in biological systems, further complicated by the intricate mechanisms of signaling and selection [29–32]. Various experimental approaches have attempted to assess the importance of TCR–antigen interactions and correlate corresponding affinities with the level of T-cell activity mediated by antigens [10,33–36]. TRAIT presents a detailed panorama of specificity and affinity, focusing on their interplay with the structural aspects of TCRs and the interfaces that they form with pMHC. As shown in Figure 2A, records of interactive TCR–antigen pairs describing antigen-specific TCR sequences are the primary source of TRAIT. A detailed annotation of the interactive pairs, including sequences of CDR3 regions, germline gene usage, sequencing methods of TCR, MHC alleles, epitope species, epitope gene of antigen, and pathologic conditions, was extracted from experimental publications. Taking mutant Kirsten rat sarcoma viral oncogene homolog (KRAS) protein as an example, TRAIT offered comprehensive knowledge of KRAS epitopes and cognate TCRs (Table S1). A total of 35 interactive entries with 6 structures and 6 affinity values were retrieved after searching with the item “Parent gene: *KRAS*” on the “Search” page, which helps with a comprehensive understanding of KRAS and rat sarcoma (RAS) drug discovery. We concluded by emphasizing how the relationships between specificity, affinity, and structure might be exploited to help provide a deeper understanding of the features of antigen recognition in T-cell immunity.

### Single-cell omics-based profiling of interactions between TCRs and antigens

Single-cell sequencing provides a powerful technique to ascertain the TCR sequences of individual T cells [37–41]. As shown in Figure 2B, TRAIT offers an omnidirectional single-cell omics map of TCRs focused on certain antigens. Detailed information on TCR–antigen pairs, including sequences of CDR3 regions and germline gene usage, is shown in a separate table. A comprehensive illustration of CDR3 sequence motifs, including interactive and non-interactive pairs, is provided and can be interactively accessed online. Highly reliable and enormous TCR–antigen pairs with both interactive and non-interactive pairs will further facilitate the development of bioinformatics algorithms [42–45] and enable sample–sample comparisons within different donors and cancer types. By incorporating non-interactive pairs, the binding characteristics of TCR–antigen pairs are illuminated in a more comprehensive perspective.

**Figure 3:**
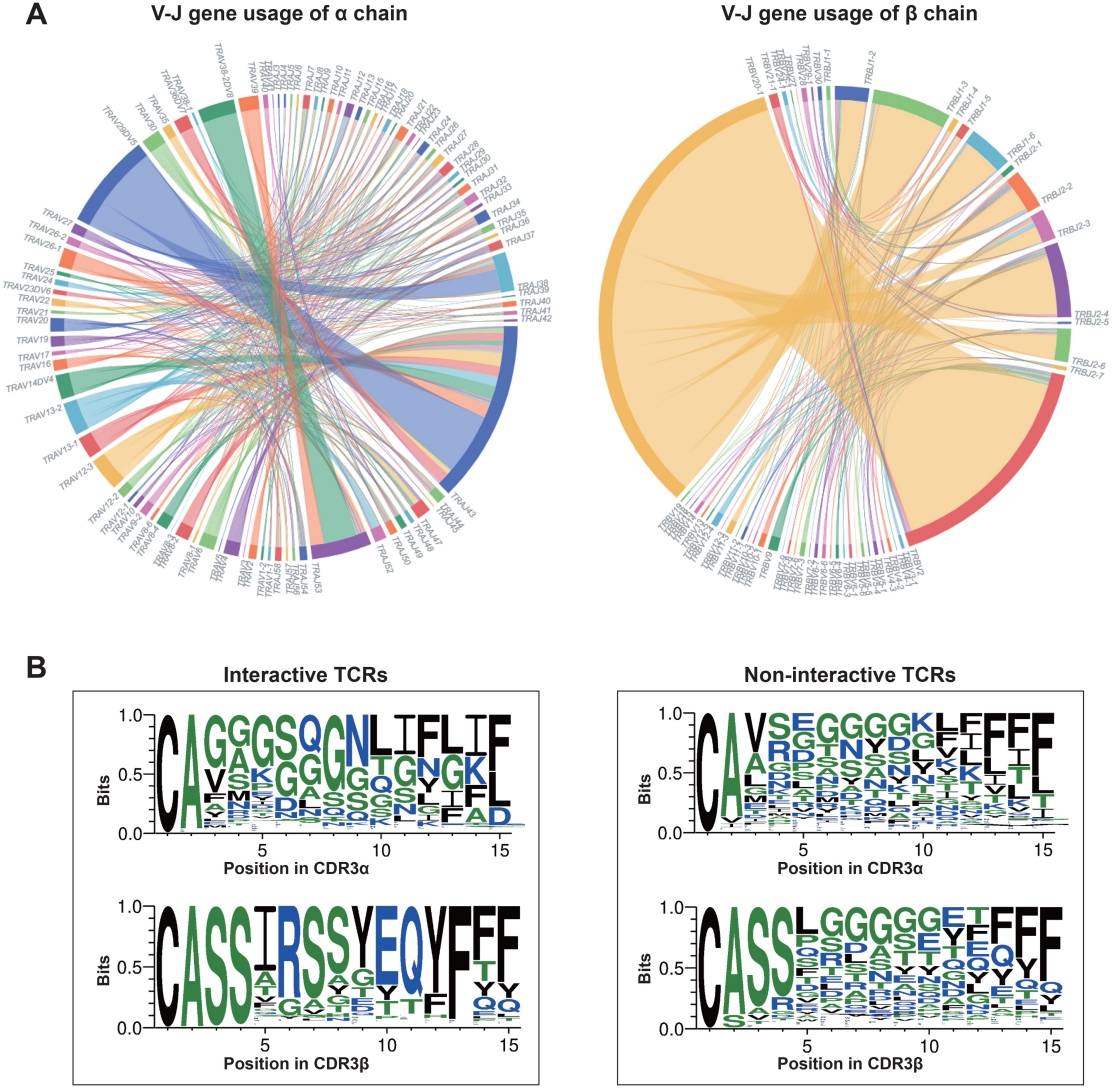
Features of TCRs related to GILGFVFTL–HLA-A*02:01 on the “Omics” page **A**. V-J gene usage of interactive TCRs. Genes with detection frequency > 5 are used for visualization. **B**. Comparison of the CDR3 motifs between interactive and non-interactive TCRs. The visualized figures for V-J genes on the web pages were generated using the D3.js tool. Sequence logos depicting interactive and non-interactive TCR–antigen pairs were generated using the WebLogo 3 tool.

Taking the peptide “GILGFVFTL” presented by HLA-A*02:01 as an example, it has 1368 entries of interactive and 65,924 non-interactive TCR records from four different donors. As shown in **Figure 3**, the amino acid residues of CDR3 are relatively different between the interactive and non-interactive TCRs related to GILGFVFTL–HLA-A*02:01. CDR3 loops of most TCRs start with cysteine and alanine, and end with phenylalanine in either interactive or non-interactive CDR3 motifs. Despite the subtle similarities, notable differences between interactive and non-interactive CDR3 motifs are observed. As shown in CDR3α of interactive TCRs, glycine and asparagine are preferred separately at positions 8 and 9, and phenylalanine shows biases at position 12. Preferences of amino acids in CDR3β are more significant. Interestingly, no significant amino acid preferences are observed for CDR3α and CDR3β for non-interactive TCRs except for the subtle enrichment of phenylalanine at positions 13 and 14.

### Thorough investigation of interactions between TCR mutations and cognate antigens

**Figure 4:**
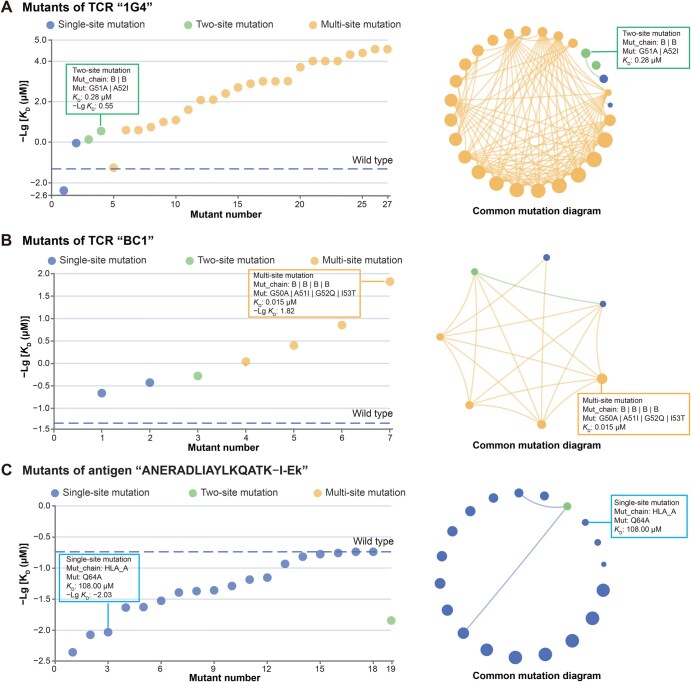
Description and visualization of binding affinities between mutations of TCR and antigens **A**. Scatter plot and circular plot illustrating interactive capacities between mutations of TCR “1G4” targeting “SLLMWITQC–HLA-A*02:01”. **B**. Scatter plot and circular plot illustrating interactive capacities between mutations of TCR “BC1” targeting “SLLMWITQC–HLA-A*02:01”. **C**. Scatter plot and circular plot illustrating interactive capacities between antigen mutations and cognate TCR “2B4”. Mutants with shared amino acid mutation sites were visualized by linking them using lines. Larger diameters of a mutant indicate higher affinities between TCRs and their corresponding antigens. ECharts was used to visualize the relationship between mutations and affinities in online TRAIT.

The manufacturing of high-affinity TCRs has been a core strategy to improve the efficacy of adoptive cell transfer therapies [46–50]. A panorama of affinities for TCR mutations and antigens was collected and systematically described in TRAIT (Figure 2C). All antigen-specific TCR mutants that have been experimentally verified are linked to measured affinities, and detailed information on mutations can be further consulted. Users may click SLLMWITQC–HLA-A*02:01 as an example, and 51 entries including 42 mutation records of TCR “1G4” and 9 mutation records of TCR “BC1”, are provided in the resulting list (Table S2). Mutants of 1G4 had affinities ranging from 0.026 nM to > 240 μM in four publications, while mutants of BC1 had an affinity of 0.015–21.4 μM with SLLMWITQC–HLA-A*02:01. Amino acid mutations in CDR regions were bolded and marked in red for easy track. Additionally, TRAIT enabled the visualization of the entire panorama through a scatter plot and a circular plot between mutations of TCRs and corresponding affinities with SLLMWITQC–HLA-A*02:01 (Figure 4A and B). Different types of mutations (including single-site mutations, two-site mutations, and multi-site mutations) are indicated in the diagram using different colored circles, with the detailed amino acid residues interactively viewed by placing the mouse on any of the circles. Consequently, mutations of TCRs with enhanced or weakened binding capacities were intuitively viewed. In summary, TRAIT is unique in providing a panorama that enables a full visualization of the mutation affinities of TCRs and is valuable for the manufacturing of high-affinity TCR profiles for research interest. Moreover, mammalian cells [51,52], yeast [53,54], and bacteriophage display [55,56] represent additional powerful, high-throughput methods capable of creating TCRs with a wide range of binding affinities, thereby providing a continuous data source for TRAIT.

### Intensive explorations of interactions between mutations of antigens and corresponding TCRs

Investigating interactions between antigens and particular TCRs will lead to a more profound understanding on immunogenicity of antigens and cross-reactivity of TCRs [57,58]. TRAIT provides the binding capacities of specific TCRs with antigens or antigen mutants. As shown in Figure 2C, mutations of antigens with the capacity interacting with TCRs are summarized in the table. Detailed information can be further queried by clicking the name of TCRs. A scatter plot and a circular plot between mutants of antigens and binding capacities with certain TCRs were drawn to directly show changes in affinities to different antigens for the same TCR. Users may click 2B4 as an example, and 25 entries including 3 wild-type records, 16 mutation records of human leukocyte antigen (HLA), and 6 mutation records of peptides, are displayed in the search list. The scatter plot and circular plot between mutants of antigens and affinities with 2B4 indicated that neither a mutant of HLA nor peptide can result in higher affinities with TCR 2B4 (Figure 4C). Linking binding affinities to mutants of antigens enables us to delve deeper into the interactions between antigens and TCRs and help us trace peptides that can better stimulate T-cell immunity and promote vaccine development.

### Clinical usage

TCR-driven therapies are introducing a new era in precision oncology treatments. As a promising therapeutic alternative, TCR-T cells can target epitopes derived from both surface and intracellular for tumor recognition [59]. Natural (wild-type) TCRs identified from tumor-infiltrating lymphocytes, circulating T cells in patients, healthy donors, or humanized mice are the main sources of TCR candidates [60]. Nonetheless, natural TCRs typically exhibit lower affinities due to thymic selection, which restricts their capacity to recognize antigens with low densities on tumor cells. To address this issue, a common strategy is to enhance the TCR’s affinity for its target [61]. Several TCRs without CDR modification have demonstrated encouraging outcomes in clinical studies for various solid tumors, as shown detailed in Table S3. Notably, an affinity-enhanced TCR-1G4 (targeted NY-ESO-1) achieved a 44% ORR among patients with metastatic melanoma [62–64], a 45%–67% ORR in individuals with metastatic synovial sarcoma, and a 80% ORR in patients with myeloma without apparent side effects [61]. Another phase 1 clinical trial indicated that TCR-T cells engineered for high affinity to MAGE-A3 antigen led to a 56% ORR in individuals with melanoma, synovial sarcoma, and esophageal cancer [65]. In this context, the information on both natural and modified TCRs provided in TRAIT is useful for the discovery of therapeutic TCR candidates for TCR-T therapies and other immunotherapeutic approaches.

## Discussion and conclusion

In this study, TRAIT is developed to provide a comprehensive TCR–antigen interaction landscape. It is unique in systematically providing TCR–antigen pairs by integrating sequences, structures, binding affinities, mutations, and clinical evaluations. More importantly, a correlation between affinity and amino acid residues is initially introduced in TRAIT to more deeply explore the interaction rules and characteristics of TCRs and antigens. Moreover, it supplies a versatile visualization platform for TCR–antigen interactions, along with an intuitive web interface for convenient data retrieval. Consequently, TRAIT will serve as an invaluable resource for researchers in both experimental and computational biologists when delving into T-cell immunology and translational medicine of immunotherapies.

Immunotherapies involving the interaction of TCRs and antigens have emerged as pivotal therapeutic strategies in oncology, with an exponentially increase expected in the future for novel TCR–antigen pairs derived from patients with different diseases [66–68]. In this regard, TRAIT will be regularly updated to provide timely data and improved web pages. Furthermore, additional annotation information encompassing detailed corresponding diseases and a focus on neoantigens, along with practical analysis tools, will be incorporated to keep pace with the rapidly evolving research landscape. These enhancements are anticipated to significantly augment the application’s efficiency within the research community. Overall, TRAIT is poised to serve as a valuable resource facilitating a comprehensive understanding of molecular mechanisms underlying antigen recognition in T-cell immunology, thereby holding immense implications for the future advancements in immunotherapeutic interventions.

## Supplementary Material

qzaf033_Supplementary_Data

## Data Availability

TRAIT is freely available at https://pgx.zju.edu.cn/traitdb to all users without any login or registration restrictions. TRAIT has been submitted to Database Commons [69] at the National Genomics Data Center (NGDC), China National Center for Bioinformation (CNCB), which is publicly accessible at https://ngdc.cncb.ac.cn/databasecommons/database/id/9707.

## CRediT author statement

**Mengmeng Wei:** Methodology, Data curation, Formal analysis, Investigation, Writing – original draft, Writing – review & editing. **Jingcheng Wu:** Methodology, Data curation, Software, Investigation. **Shengzuo Bai:** Software, Investigation, Visualization, Writing – review & editing. **Yuxuan Zhou:** Data curation, Investigation. **Yichang Chen:** Investigation, Visualization. **Xue Zhang:** Visualization. **Wenyi Zhao:** Methodology, Visualization. **Ying Chi:** Investigation. **Gang Pan:** Supervision. **Feng Zhu:** Conceptualization, Methodology. **Shuqing Chen:** Conceptualization, Funding acquisition. **Zhan Zhou:** Conceptualization, Funding acquisition, Supervision, Writing – review & editing. All authors have read and approved the final manuscript.
