# Supplementary material for: TRAIT: A Comprehensive Database for T-cell Receptor–antigen Interactions
Source: Genomics Proteomics Bioinformatics. 2025 Apr 21;23(3):qzaf033. doi: 10.1093/gpbjnl/qzaf033 (PMC12448929; doi:10.1093/gpbjnl/qzaf033)
Supplement: qzaf033_Supplementary_Data [file qzaf033_supplementary_data.zip › supplementary material captions.docx]

**Supplementary material**

**Table S1** **Result list by searching with “Parent gene: *KRAS*” in TRAIT**

**Table S2** **List of TCR mutations targeting SLLMWITQC**–**HLA-A*02:01**

**Table S3** **List of representative TCRs on clinical trials**
